# Mind the wind: microclimate effects on incubation effort of an arctic seabird

**DOI:** 10.1002/ece3.1988

**Published:** 2016-02-21

**Authors:** Christoffer Høyvik Hilde, Christophe Pélabon, Loreleï Guéry, Geir Wing Gabrielsen, Sébastien Descamps

**Affiliations:** ^1^ Department of BiologyNorwegian University of Science and Technology7491TrondheimNorway; ^2^ Centre for Biodiversity DynamicsDepartment of BiologyNorwegian University of Science and Technology7491TrondheimNorway; ^3^ Département de BiologieChimie et GéographieUniversité du Québec à RimouskiRimouskiCanada; ^4^ Fram CentreNorwegian Polar Institute9296TromsøNorway

**Keywords:** Climate change, common eider, incubation, life‐history, microclimate, nest shelter, nest site selection, wind

## Abstract

The energetic costs of reproduction in birds strongly depend on the climate experienced during incubation. Climate change and increasing frequency of extreme weather events may severely affect these costs, especially for species incubating in extreme environments. In this 3‐year study, we used an experimental approach to investigate the effects of microclimate and nest shelter on the incubation effort of female common eiders (*Somateria mollissima*) in a wild Arctic population. We added artificial shelters to a random selection of nesting females, and compared incubation effort, measured as body mass loss during incubation, between females with and without shelter. Nonsheltered females had a higher incubation effort than females with artificial shelters. In nonsheltered females, higher wind speeds increased the incubation effort, while artificially sheltered females experienced no effect of wind. Although increasing ambient temperatures tended to decrease incubation effort, this effect was negligible in the absence of wind. Humidity had no marked effect on incubation effort. This study clearly displays the direct effect of a climatic variable on an important aspect of avian life‐history. By showing that increasing wind speed counteracts the energetic benefits of a rising ambient temperature, we were able to demonstrate that a climatic variable other than temperature may also affect wild populations and need to be taken into account when predicting the effects of climate change.

## Introduction

Most scenarios of future climate predict a further increase in ambient and seawater temperatures, precipitation, and in the frequency of extreme weather events (Christensen et al. 2013). Although rising temperatures often have a negative effect on life‐history traits and population dynamics (Both et al. 2006; Drever et al. 2012), some species may benefit from increasing temperatures (McKinnon et al. 2013). Incubation is a demanding phase of avian life‐history susceptible to changes in thermal conditions (Reid et al. 2000), and a milder climate may decrease the energetic costs during this reproductive phase (D'Alba et al. 2009). This effect may be even more pronounced for birds incubating in extreme environments (Tulp and Schekkerman 2006). For instance, Arctic breeding shorebirds experience a daily energy expenditure up to 50% higher than birds breeding in temperate areas, the energy expenditure being highest during incubation (Piersma et al. 2003). Even though rising ambient temperatures may decrease the energetic costs of incubation, other climatic factors such as wind and humidity may have the opposite effect. Wind can increase the rate of heat loss by disrupting the plumage and reducing thermal insulation, leading to an increase in energy expenditure (Weimerskirch et al. 2002). Even small changes in wind speed can drastically increase the convection of heat from the incubating bird to the environment (Heenan and Seymour 2012). Similarly, optimal nest humidity is important for successful chick development and hatching (Ar and Rahn 1980), and rainfall can negatively affect the survival of both chicks (Anctil et al. 2014) and parents (Öberg et al. 2015). More humid conditions during incubation could possibly increase the energy spent for maintaining an optimal body temperature. To our knowledge, however, the effects of neither wind nor relative humidity on the energetic costs of incubation have been investigated in birds.

The amount of shelter provided by a nest could potentially reduce the energy required by an incubating bird for maintaining body and clutch temperature at an optimal level and thus reduce the incubation costs. Hence, parents occupying sheltered nest sites may have better breeding performance than those occupying exposed nest sites, or similar breeding performance, but at a lower energetic cost, these effects being more pronounced during years with adverse weather conditions (e.g., strong wind, cold temperature, and precipitation). Studies have shown that female common eiders (*Somateria mollissima*) nesting on a windswept island lose body weight faster than those in more sheltered colonies (Kilpi and Lindstrom 1997) and that artificial shelters may decrease mass loss during incubation (Fast et al. 2007) independently of the female phenotypic quality (D'Alba et al. 2009). Although these studies confirm the important role of ambient temperature on the energetic costs of incubation, they did not investigate the specific effects of other microclimatic factors, such as wind and humidity, and their possible interaction effect on these costs.

If we are to predict the impact of climate change on bird populations, it is crucial to understand the causal relationships between microclimate and incubation effort. Examining the direct and combined effects of wind, humidity and ambient temperature on the energy expenditure during incubation may help understand the effects of microclimate on incubation effort. However, the effects of nest site characteristics on incubation effort may be confounded with variation in individual quality. D'Alba et al. (2009) found that common eider females with naturally sheltered nest sites produced larger clutches than nonsheltered females, implying that females of better quality preferred naturally sheltered nest sites. Consequently, an experimental approach (as in D'Alba et al. (2009)) is strongly recommended for such a study in order to control for the covariance between individual heterogeneity and nest quality (Wilson and Nussey 2010).

We investigated the effects of wind, ambient temperature, and humidity on incubation effort of females in an Arctic population of common eiders in Kongsfjorden, Svalbard. Using the body mass loss during incubation as an index of incubation effort, we predicted that an increase in wind and humidity would increase the incubation effort, while an increase in temperature would lower the energy required during incubation and thus lower the incubation effort. We ran the study over 3 years, allowing us to test for interannual variations, expecting female eiders nesting in years with more adverse weather conditions to have a higher incubation effort compared to female eiders nesting in milder years. In order to disentangle the specific effect of wind from the effects of other microclimatic variables on the incubation effort, while controlling for variation in female quality, we experimentally manipulated the degree of wind protection of the nest by adding artificial shelters around a random selection of nests occupied by incubating females. Female eiders with a nest shelter were expected to have a lower incubation effort than those occupying nonsheltered nests, especially in years with high wind speeds.

## Methods

### Study species and study site

The common eider is a sea duck known to be sensitive to climatic conditions (Lehikoinen et al. 2006; Descamps et al. 2010). It has a circumpolar distribution breeding mainly in Arctic and Boreal marine areas. Female eiders lay eggs in small cup‐shaped holes filled with down and they incubate without male aid, relying upon accumulated body reserves during the whole incubation period of ca. 25 days (Hanssen et al. 2002). During this period, the females occasionally leave the nest for a short trip to nearby water to drink (Criscuolo et al. 2000). On Svalbard, incubating females generally nest on small barren islands and lose approximately 35–40% of their initial body weight during incubation (Gabrielsen et al. 1991). This body mass loss during incubation is a good proxy of the incubation effort for this species.

This study was conducted on Prins Heinrich island outside Ny‐Ålesund in Kongsfjorden (78°55′N, 12°00′E), Svalbard, during three subsequent breeding seasons (2012–2014). This island (2.43 hectares) is covered with tundra vegetation and soil, with a surrounding shoreline of small rocks. Between 200 and 400 eiders nest on the island (*N* = 218, 271, and 362 in 2012, 2013, and 2014, respectively), as well as a few pairs of Barnacle geese (*Branta leucopsis*), Glaucous gulls (*Larus hyperboreus*), and Arctic terns (*Sterna paradisaea*). The main predator of common eider eggs is the Glaucous gull, of which three pairs were nesting on the island each year. In this population, females started laying eggs in early June (4 June 2012 and 2013 and 3 June 2014).

### Nest‐site assessment

All nests on the island were marked and numbered with a wooden stick placed into the ground close to the nest. Nesting birds were monitored every second day until incubation started, and the number of eggs per nest was recorded at each visit. Female eiders usually lay 3–6 eggs and start incubation before the last egg is laid (Hanssen et al. 2002). Nest sites placed close to rocks, driftwood, or natural cavities provided some apparent degree of shelter. However, preliminary analyses showed no marked effect of those natural shelters on incubation effort (mean ± SE daily mass loss: with natural shelter = 1.32% d^−1^ ± 0.032; without natural shelter = 1.31% d^−1^ ± 0.031; See Appendix S2 for statistical tests). Thus, we pooled the data from all nonmanipulated nests into a single category, referred to as “nonsheltered” for further analyses.

### Recorded variables

Females which had finished egg‐laying and started incubating were captured using a fishing rod with a nylon loop at the end. Birds were weighed to the nearest 5 g using a Pesola scale, their tarsus length and head‐bill length were measured using a caliper, and wing length was measured using a ruler to the nearest mm. The mean dates for the first captures were 12 June 2012 and 2013, and 14 June 2014. After 15 (min 13, max 17) days, the birds were recaptured and weighed. From the two measurements of body mass, we calculated the percentage decrease in body mass per day as: $\%\;\text{daily mass loss}=100\times \frac{\left(\text{Initial mass}\;-\;\text{final mass}\right)}{\left(\text{Initial mass}\;\times \;\text{number of days}\right)}$, where number of days refers to the period between the first and second capture. This measure was used as a proxy for the incubation effort. The nests were not monitored between the first and second capture so all females were only disturbed twice during our study. Body mass has been shown to be a good proxy of body condition in common eiders (Descamps et al. 2010), and correcting for structural size did not affect the results in this study (not shown).

**Figure 1:**
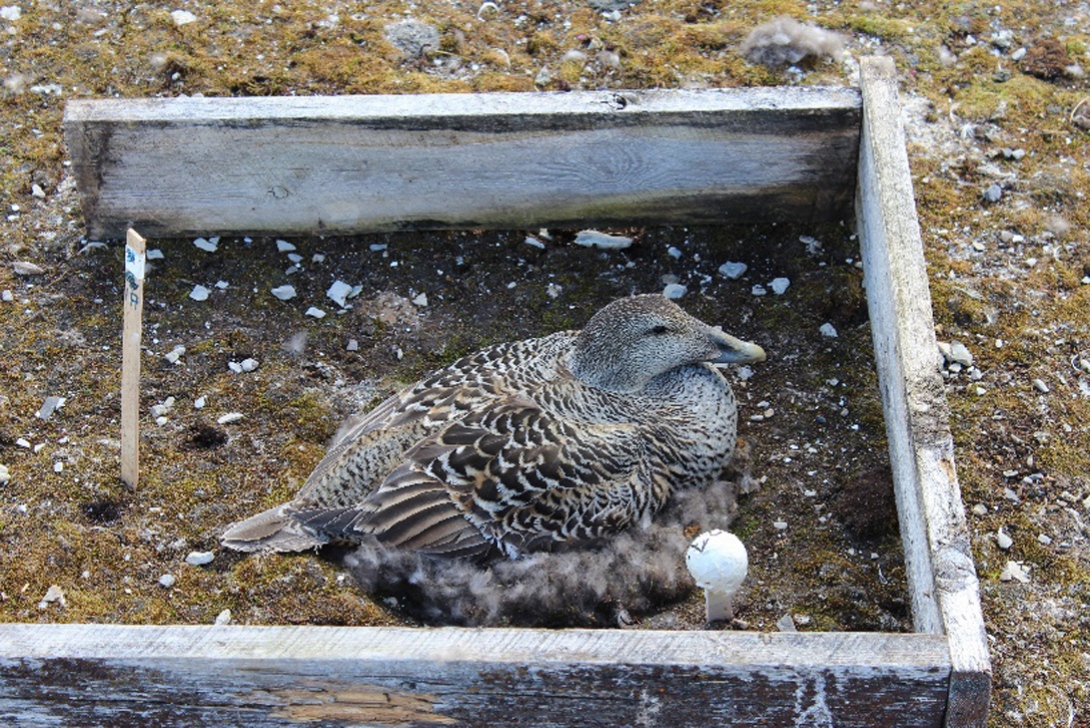
Female common eider (*Somateria mollissima*) with an artificial shelter and the temperature/humidity logger inside. A white plastic ball with air vents was placed around the logger to prevent exposure to direct sunlight. Photo: Elise Skottene.

At first capture, a temperature and humidity logger (iButton Hydrocron DS1921—Maxim Integrated Products, Sunnyvale, CA, USA) was placed approx. 10 cm from the edge of each nest at eider head height (Fig. 1). Ambient temperature and humidity were logged every 10 min until the logger was retrieved at the second capture. In 2014, an anemometer (Davis Instruments, Hayward, CA, USA), logging wind speed and direction, was placed on the island and the wind data were recorded during the whole study period. Mean wind speed measured on the island in 2014 was 2.77 m/s (SD = 0.29) with a mean wind direction of 203.4 degrees (SD = 79.1). Wind speed measurements on the island were highly correlated with those logged by the Ny‐Ålesund weather station located 1.3 km from the study site (*r* = 0.98). Hence, we used the wind speed data from Ny‐Ålesund to estimate the wind on the island during the 3 years of the study.

### Nest shelter experiment

To examine the effect of the wind on incubation effort while controlling for potential variation in female quality and keeping humidity and ambient temperature unchanged, we randomly assigned artificial shelters to females with nonsheltered nests. The artificial shelters were placed during the first capture and consisted of three wooden planks (*c*. 15 cm height, 50 cm length) protecting three sides around the nest (Fig. 1) allowing the bird to move freely in and out of the nest site. A total of 11 and 17 nests were provided with such shelters in 2013 and 2014, respectively. Due to logistic constraints, no artificial shelters were added in 2012. Neither ambient temperature nor humidity was affected by the presence of the artificial shelters (Appendix S2). The wind‐shield effect of the artificial shelters was confirmed by using a handheld anemometer (Mastech, Guangdong, China) to measure the wind inside the shelters. After placing a shelter around a nest, the female was observed from a distance until she returned to the nest to make sure the shelter was accepted and the nest was not predated. All females returned to the nest within approx. 10 minutes.

### Statistical analyses

To test for potential differences in clutch size and body mass at first capture between nest shelter categories, we conducted ANOVAs including both shelter category and year as predictor variables. Data from 2012 were excluded from these analyses because no artificial shelters were used during this year.

We tested the effect of nest shelter on incubation effort, using an ANOVA with shelter category, year and their interaction as predictor variables. To further examine the effects of microclimate variables on the incubation effort of sheltered versus nonsheltered females, we performed two separate multiple regressions, one for each shelter category, because wind was absent in artificially sheltered nest sites. In a first model, we tested the effect of microclimate on the incubation effort of nonsheltered females, with average wind speed, ambient temperature, humidity, and year as predictor variables. In the second model, for artificially sheltered females, only ambient temperature, humidity, and year were used as predictor variables. All covariates were mean centered in both analyses. Model selection was performed using the Akaike's information criterion corrected for small sample size (AICc, Burnham and Anderson (2002)). When several models were within 2 AICc units of the model with the lowest AICc value, we performed model averaging on all these models to obtain weighted parameter estimates (Burnham and Anderson 2002; Nakagawa and Freckleton 2011). The weighted parameter estimates were calculated using full‐model averaging (i.e., models not containing the variable of interest contribute zero to the calculation of the average parameter estimate), which is recommended in case of high model selection uncertainty (Symonds and Moussalli 2011). Distributions of the residuals were inspected for all models and confirmed that no transformation was necessary to achieve normality or homoscedasticity. All statistical analyses were performed using R v.3.1.2 (R Core Team, 2013).

## Results

The mean body weight of incubating common eiders at first capture did not differ between years and between shelter categories (Table 1, Appendix S2). The mean clutch size was not different among shelter categories but tended to vary among years, the average clutch size being larger in 2012 (Table 1, Appendix S2).

**Table 1:** Mean (±SE) values of female body mass, clutch size at the start of the incubation and the three microclimate variables for each year and shelter category

	Mass (g)	Clutch Size	Wind (m/s)	Temp (°C)	Humidity (%RH)
2012 (*N* = 20)	1803 ± 19.1	4.2 ± 0.12	2.63 ± 0.02	8.10 ± 0.10	74.97 ± 0.91
2013 (*N* = 24)	1820 ± 24.0	3.5 ± 0.21	3.35 ± 0.07	5.58 ± 0.18	87.63 ± 0.53
2014 (*N* = 43)	1823 ± 14.6	3.0 ± 0.16	2.59 ± 0.07	6.21 ± 0.15	74.29 ± 0.57
Nonsheltered (*N* = 63)	1812 ± 11.8	3.56 ± 0.12	2.83 ± 0.06	6.57 ± 0.17	77.16 ± 0.82
Artificially sheltered (*N* = 24)	1833 ± 23.0	3.10 ± 0.25	2.93 ± 0.11	6.21 ± 0.17	80.66 ± 1.48

**Figure 2:**
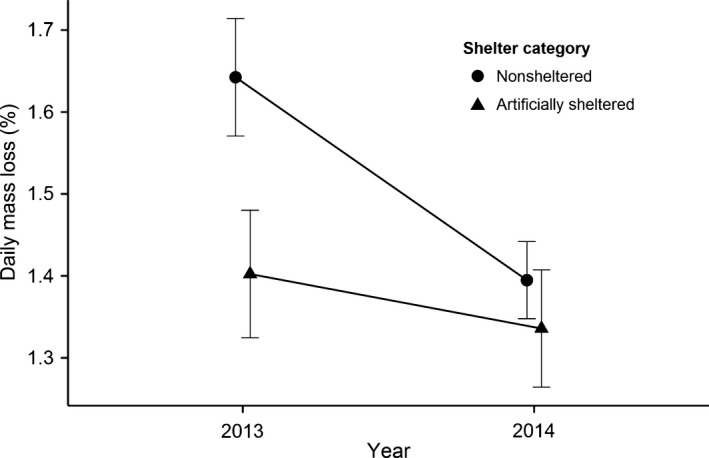
Difference in incubation effort as measured by daily mass loss (%) in common eider females between the two shelter categories and between years. Mean ± SE are obtained from the best model presented in Table 2.

Females without artificial shelter had a higher incubation effort in 2013 compared to 2014 (Table 2; Fig. 2). This was most likely due to the more challenging conditions encountered by the birds in 2013, with stronger wind, colder temperature, and higher humidity (Table 1). Females with an artificial shelter had a lower incubation effort than those without shelter, and this difference was more pronounced in 2013 (Fig. 2).

**Table 2:** Model selection for the effects of nest shelter (no shelter vs. artificial shelter) and year (2013 and 2014) on the incubation effort as measured by the daily mass loss (%) of incubating female common eiders. K is the number of parameters estimated, AICc the Aikake information criterion corrected for small sample size, ∆AICc is the difference in AICc compared to the model with lowest AICc, wAICc is the AICc weights, and *R* ^2^ is the fraction of variance explained by the model

Effect of nest shelter and year on daily mass loss (%)					
Predictors	*K*	AICc	∆AICc	wAICc	*R* ^2^
Shelter category × Year	6	−77.1	0	0.916	0.41
Shelter category + Year	5	−72.3	4.78	0.084	0.35
Year	3	−61.3	15.83	0.000	0.21
Shelter category	4	−52.2	24.9	0.000	0.09
Intercept only	2	−48.0	29.14	0.000	0.00

**Figure 3:**
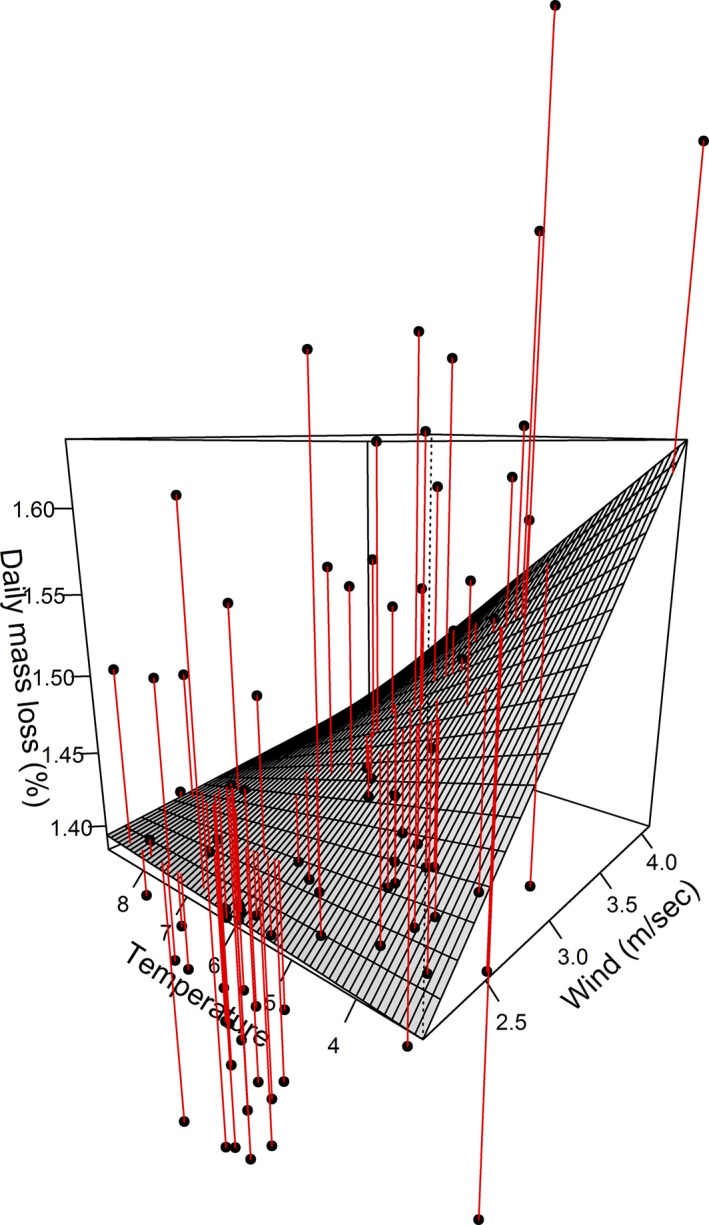
Estimated effects of ambient temperature and wind speed on the incubation effort as measured by daily mass loss (%) of nonsheltered common eider females. The figure is made using parameter estimates presented in Table 4.

The estimated effects of the microclimatic variables on nonsheltered females were obtained using model averaging. For these females, higher wind speeds increased incubation effort (Tables 3 and 4). Differences in ambient temperature had little effect on incubation effort at low wind speeds, but became important when wind speed increased (Table 4; Fig. 3). Relative humidity had no marked effect on incubation effort over the range of humidity observed. In artificially sheltered females, neither ambient temperature nor humidity had an effect on incubation effort (Table 5), and the incubation effort was similar in 2013 and 2014, despite marked differences in ambient temperature and humidity. These results confirm the importance of the wind as microclimatic factor affecting incubation effort in common eider females.

**Table 3:** Model selection for the effects of microclimate and year (2012, 2013, and 2014) on the incubation effort as measured by daily mass loss (%) of nonsheltered common eider females. Only models within 2 units of AICc are shown

Predictors	*K*	AICc	∆AICc	wAICc	*R* ^2^
Wind + Year	5	−81.2	0	0.37	0.53
Wind × Temp + Year	7	−80.9	0.29	0.32	0.57
Temp + Year	5	−79.6	1.57	0.17	0.52
Humidity + Temp + Year	6	−79.4	1.82	0.15	0.54

**Table 4:** Effects of microclimate and year on daily mass loss for nonsheltered females. All explanatory variables are mean centered. Weighted averages of the parameter estimates were calculated using all models within 2 AICc units of the model with the lowest AICc value (Table 4) (see Appendix S2 for complete model selection). The parameter estimates were calculated using the full‐model averaging method (Symonds and Moussalli 2011)

Parameter	Estimate ± SE	Relative importance
Intercept (2012)	1.334 ± 0.044	
Year (2013)	0.246 ± 0.068	1
Year (2014)	0.056 ± 0.058	1
Wind	0.061 ± 0.064	0.68
Temp	−0.013 ± 0.024	0.63
Temp × Wind	−0.019 ± 0.033	0.32
Humidity	−0.0008 ± 0.002	0.15

**Table 5:** Model selection for the effects of microclimate and year (2013 and 2014) on the daily mass loss (%) of sheltered females

Predictors	*K*	AICc	∆AICc	wAICc	*R* ^2^
Intercept	2	−23.9	0	0.48	0.00
Year	3	−22.8	1.1	0.27	0.06
Humidity	3	−22.6	1.32	0.25	0.05
Temperature	3	−21.6	2.35	0.11	0.01
Temperature + Year	4	−20.7	3.23	0.07	0.09
Temperature + Humidity	4	−20.4	3.50	0.06	0.08

## Discussion

This study shows that microclimate has a strong effect on the incubation effort of female common eiders, but mostly for females incubating in exposed nests (Fig. 2). For these females, an increase in wind speed increased the incubation effort while increasing ambient temperatures tended to counteract this effect (Fig. 3). When protected from the wind, the influence of temperature on the incubation effort was limited. Consequently, the beneficial effects of sheltered nests were strongly variable from year to year, depending on the weather conditions. In 2013, artificially sheltered females lost on average 0.24% less mass each day compared to nonsheltered females (ca. 77 g in total during 25 days of incubation), and their incubation effort was not affected by either ambient temperature or humidity. In contrast, in 2014 when the wind speed was on average 1 m/s lower than in 2013, the difference in incubation effort between shelter categories was less pronounced, and artificially sheltered females lost only 0.06% less mass each day compared to nonsheltered females (ca. 19 g in total during 25 days of incubation). D'Alba et al. (2009) showed that exposed nests had lower nest site temperatures than sheltered nests at high wind speeds (> 5 m/s). Our study suggests that even small changes at relatively low wind speeds (all the wind speeds recorded were < 4 m/s) can markedly increase the incubation effort of female eiders. An increase in wind speed of 1 m/s, at the average temperature, increased the daily mass loss by 0.062% per day.

Absorption of solar radiation by the plumage of an incubating bird may increase its body temperature (Bakken and Angilletta 2014) and thus decrease the energy required during incubation. It has been shown that birds exposed to solar radiation have a lower metabolic rate than nonexposed birds (Wolf and Walsberg 1996). However, the positive effect of radiation was shown to decrease with an increase in wind speed (Wolf and Walsberg 1996). This could partly explain the interannual differences in body mass loss for the nonsheltered birds. The warming by solar radiation might have had a more positive effect on the incubation energetics of the females incubating in 2012 and 2014, which experienced lower wind speeds (Table 1) and likely more solar radiation (CHH and SD, personal observation) compared to 2013. Although solar radiation could be a confounder, it is unlikely that the effect of wind reported in this study was affected by it. Because the shelters used in this study did not have a roof, both females with and without nest shelter were exposed to the same level of radiation.

Unlike D'Alba et al. (2009), we were unable to detect any differences in incubation effort between females with different degrees of natural shelter. A likely explanation is that the natural shelters included in our study (i.e., rocks, piece of wood) offered limited protection from the wind. A few nest sites with an apparently higher degree of natural shelter were available on the island, but we were unable to capture the females occupying these nest sites and we could not include them in our study. Nevertheless, our results indicate that by choosing a well sheltered nest site, female common eiders could reduce a large part of the negative impact of wind on incubation energetics. Still, naturally sheltered nest sites were not preferred over nonsheltered nest sites by early laying females (CHH, personal observation), and many females chose nonsheltered nest sites even if sheltered ones were available. This suggests that breeding in a sheltered nest may also have some costs. Predation is often the main cause of reproductive failure in birds (Martin 1993) and nest site selection may represent a trade‐off between predation risk and appropriate microclimate for incubation (Amat and Masero 2004). Öst and Steele (2010) have reported that predation risk in common eiders increased with nest shelter, providing a plausible explanation for the observed lack of selection for sheltered nest sites by the females in our study. Moreover, some sheltered nest sites in our study area were close to the shore where the risk of being flooded was high. The energetic benefit from a sheltered nest site may thus be counterbalanced by a higher fitness cost, in terms of nest predation and/or flood risk (Viera et al. 2006). Future climate change may skew this trade‐off if wind speeds in the Arctic increases, and thereby increases the benefits of more sheltered nest sites.

Surprisingly, little is known about the effects of climate change on surface winds (Christensen et al. 2013). However, a recent metastudy by Sydeman et al. (2014) reported an increased likelihood of wind intensification toward higher latitudes. Our study suggests that such an increase in wind speed would counteract the energetic gain from the rising ambient temperature predicted from climate change and could possibly increase the energetic costs of incubation, although only at exposed nest sites. These results emphasize the importance of wind, in interaction with ambient temperature, as a key environmental factor that should be accounted for when predicting the effects of a changing climate on breeding populations of ground nesting birds in the Arctic. Most of the documented effects of climate change on seabirds are indirect effects such as changes in food resources or foraging performance (Jenouvrier 2013). However, direct effects of climate change on life‐history traits, such as timing of breeding (Visser et al. 2009), breeding success (Descamps et al. 2015), or reproductive effort (Öberg et al. 2015) should not be overlooked. We show that to fully understand the consequences of climate change on the life‐histories of breeding birds in the Arctic, studies are needed to investigate whether wind patterns in the Arctic are likely to change, and how such changes may affect bird species with different breeding strategies.

## Data accessibility

The data will be accessible on DRYAD prior to publication.

## Conflict of Interest

None declared.

## Supporting information


**Appendix S1.** Correlations between microclimatic variables and biometrical measurements.

**Appendix S2.** Complete model selection tables.
