# Supplementary material for: Mind the wind: microclimate effects on incubation effort of an arctic seabird
Source: Ecol Evol. 2016 Feb 21;6(7):1914–21. doi: 10.1002/ece3.1988 (PMC4831427; doi:10.1002/ece3.1988)
Supplement: Supplementary file 1 — Appendix S1. Correlations between microclimatic variables and biometrical measurements. [file ECE3-6-1914-s001.docx]

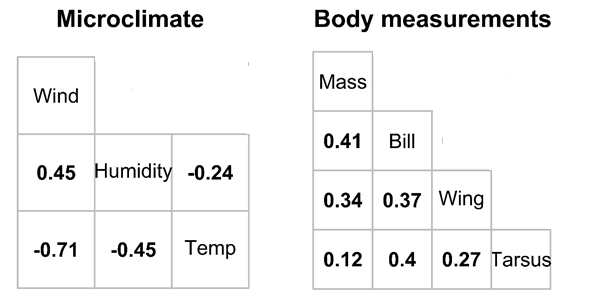


Figure 1. Pearson correlations between i) microclimate variables (upper right values are between artificially sheltered females and lower left is between non-sheltered females) and ii) body measurements. Wind was not present with artificially sheltered females, thus no correlations with wind is shown.
