# Supplementary material for: Mind the wind: microclimate effects on incubation effort of an arctic seabird
Source: Ecol Evol. 2016 Feb 21;6(7):1914–21. doi: 10.1002/ece3.1988 (PMC4831427; doi:10.1002/ece3.1988)
Supplement: Supplementary file 2 — Appendix S2. Complete model selection tables. [file ECE3-6-1914-s002.docx]

Table 1). Model selection of ANOVAs to test the effects of nest-shelter (natural shelter vs. no shelter) and year (2013 and 2014) on the daily mass loss (%) of incubating female eiders. K is the number of parameters estimated, AICc the Aikake information criterion corrected for small sample size, ∆AICc is the difference in AICc compared to the model with lowest AICc, wAICc is the AICc weights and R^2^ is the fraction of variance explained by the model (R^2^ = 1- SSresid/SStot).

| Predictors | K | AICc | ∆AICc | wAICc | R^2^ |
| --- | --- | --- | --- | --- | --- |
| Year | 4 | -78.5 | 0 | 0.754 | 0.50 |
| Shelter category+Year | 5 | -76.3 | 2.24 | 0.246 | 0.50 |

Table 2). Model selection for the effects of nest-shelter (artificial shelter vs. no shelter) on the ambient temperature (˚C) by the nest of incubating female eiders.

| Predictors | K | AICc | ∆AICc | wAICc | R^2^ |
| --- | --- | --- | --- | --- | --- |
| Only intercept | 2 | 191.9 | 0 | 0.499 | 0.00 |
| Shelter category | 4 | 192.1 | 0.2 | 0.501 | 0.03 |

Table 3). Model selection for the effects of nest-shelter (artificial shelter vs. no shelter) on the relative humidity (%RH) by the nest of incubating female eiders.

| Predictors | K | AICc | ∆AICc | wAICc | R^2^ |
| --- | --- | --- | --- | --- | --- |
| Only intercept | 2 | 459.2 | 0 | 0.499 | 0.00 |
| Shelter category | 4 | 459.6 | 0.4 | 0.501 | 0.01 |

Table 4). Model selection for the effects of nest-shelter (no shelter vs. Artificial shelter) and year on the body weight at first capture.

| Predictors | K | AICc | ∆AICc | wAICc | R^2^ |
| --- | --- | --- | --- | --- | --- |
| Only intercept | 2 | 1050 | 0 | 0.389 | 0.00 |
| Shelter category | 3 | 1051 | 1.32 | 0.201 | 0.01 |
| Year | 3 | 1052 | 1.68 | 0.168 | 0.01 |

Table 5). Model selection for the effects of nest-shelter (no shelter vs. Artificial shelter) and year on the clutch size.

| Predictors | K | AICc | ∆AICc | wAICc | R^2^ |
| --- | --- | --- | --- | --- | --- |
| Year | 3 | 196 | 0 | 0.584 | 0.05 |
| Only intercept | 2 | 197 | 1.36 | 0.296 | 0.00 |
| Shelter category | 3 | 199 | 3.16 | 0.120 | 0.01 |

Table 6). Model selection for the effects of nest-shelter (no shelter vs. artificial shelter) and year (2013 and 2014) on the daily mass loss (%) of incubating female eiders.

| Predictors | K | AICc | ∆AICc | wAICc | R^2^ |
| --- | --- | --- | --- | --- | --- |
| Shelter category*Year | 6 | -77.1 | 0 | 0.916 | 0.41 |
| Shelter category + Year | 5 | -72.3 | 4.78 | 0.084 | 0.35 |
| Year | 3 | -61.3 | 15.83 | 0.000 | 0.21 |
| Shelter category | 4 | -52.2 | 24.9 | 0.000 | 0.09 |

Table 7). Model selection for the effects of microclimate and year (2012, 2013 and 2014) on the daily mass loss (%) of non-sheltered females.

| Predictors | K | AICc | ∆AICc | wAICc | R^2^ |
| --- | --- | --- | --- | --- | --- |
| Wind + Year | 5 | -81.2 | 0 | 0.37 | 0.53 |
| Wind:Temp + Year | 7 | -80.9 | 0.29 | 0.32 | 0.57 |
| Temp + Year | 5 | -79.6 | 1.57 | 0.17 | 0.52 |
| Humidity + Temp + Year | 6 | -79.4 | 1.82 | 0.15 | 0.54 |
| Humidity + Wind + Year | 6 | -79.4 | 2.16 | 0.07 | 0.53 |
| Wind + Temp + Year | 6 | -78.8 | 2.35 | 0.06 | 0.53 |
| Year | 4 | -78.5 | 2.64 | 0.05 | 0.49 |
| Wind + Temp + Humidity + Wind:Temp + Year | 8 | -78.3 | 2.84 | 0.05 | 0.57 |
| Temp:Humidity + Year | 7 | -77.3 | 3.85 | 0.03 | 0.54 |
| Humidity + Year | 5 | -77.1 | 4.03 | 0.03 | 0.50 |
| Wind + Temp + Humidity + Year | 7 | -77.1 | 4.08 | 0.03 | 0.54 |
| Wind + Temp + Humidity + Wind:Humidity + Wind:Temp + Year | 9 | -76.8 | 4.40 | 0.02 | 0.58 |
| Wind : Humidity + Year | 7 | -76.5 | 4.70 | 0.02 | 0.54 |
| Wind + Temp + Humidity + Wind:Temp | 6 | -75.7 | 5.47 | 0.01 | 0.51 |

Table 8). Model selection for the effects of microclimate and year (2013 and 2014) on the daily mass loss (%) of sheltered females.

| Predictors | K | AICc | ∆AICc | wAICc | R^2^ |
| --- | --- | --- | --- | --- | --- |
| Intercept | 2 | -23.9 | 0 | 0.48 | 0.00 |
| Year | 3 | -22.8 | 1.1 | 0.27 | 0.06 |
| Humidity | 3 | -22.6 | 1.32 | 0.25 | 0.05 |
| Temp | 3 | -21.6 | 2.35 | 0.1 | 0.01 |
| Temp + Year | 4 | -20.7 | 3.23 | 0.06 | 0.09 |
| Temp + Humidity | 4 | -20.4 | 3.5 | 0.06 | 0.08 |
| Temp : Humidity | 5 | -20.2 | 3.68 | 0.05 | 0.19 |
| Humidity + Year | 4 | -19.9 | 4.00 | 0.04 | 0.06 |
| Temp:Humidity + Year | 6 | -18.2 | 5.73 | 0.02 | 0.24 |
| Temp + Humidity + Year | 5 | -17.5 | 6.45 | 0.01 | 0.09 |
